# Adequacy of prenatal care services and associated factors in Southern Ethiopia

**DOI:** 10.1186/s13690-021-00614-3

**Published:** 2021-06-07

**Authors:** Afework Tadele, Bekelu Teka

**Affiliations:** Population and Family Health, Jimma University, Jimma, Ethiopia

**Keywords:** Adequacy, PMA, Prenatal care, Southern Ethiopia

## Abstract

**Background:**

Prenatal care is an important component for the continuum of care in maternal and child health services. Despite increased attention on prenatal care service coverage, the adequacy of service provision has not been well addressed in Ethiopia. Therefore, this study aimed to describe the status of the adequacy of prenatal care and its associated factors in Southern Ethiopia.

**Method:**

A longitudinal study done by the Performance care Monitoring and Accountability (PMA2020) project was used. The study was conducted from August 2016 to January 2017 in Southern Ethiopia. A multistage stratified cluster design in which all enumeration areas were randomly selected using probability proportional to size and all households were screened to identify 324 pregnant women of six or more months. Questions regarding early attendance of prenatal care, enough visits, and sufficient services were asked to measure the adequacy of prenatal care. Finally, an ordered logistic regression analysis was employed to assess factors associated with the adequacy of prenatal care services.

**Results:**

Of the total pregnant women 44.21 % attended enough visits, 84.10 % had early visits, and 42.03 % received sufficient services. The women residing in urban areas had 2.35 odds of having adequate prenatal care in reference to rural areas (adjusted odds ratio (aOR) 2.35 [95 % CI 1.05–5.31]). Women who attended primary and secondary education had 2.42(aOR 2.42 [95 % C.I. 1.04, 5.65]), and 4.18 (aOR 4.18 [95 % CI 1.32, 13.29]) odds of adequate prenatal care in reference with those who never attended education respectively. The women participating in one to five networks have 2.18 odds of adequate prenatal care in reference to their counterparts (aOR 2.78 [95 % CI 1.01, 7.71]).

**Conclusions:**

The adequacy of prenatal care services in Southern Ethiopia is very low. The Ethiopian health care system should strengthen one to five networks to discuss on family health issues. Further research, should validate the tools and measure the adequacy of the services in different contexts of Ethiopia using a mixed method study for an in-depth understanding of the problem.

## Background

High-quality care throughout pregnancy has a lion share contribution to the continuum of maternal, newborn and child health care. This illustrates that boosting the health of mothers and their born requires but is not limited to the delivery of health services by skilled health professionals during the antenatal period [1–3]. Prenatal care services provide an opportunity to promote healthy behaviors during pregnancy, identify and treat health problems, and raise awareness of danger signs that may occur during pregnancy [4–7]. Essentially, to offer the interventions recommended by the World Health Organization (WHO), it is crucial to guarantee universal coverage of health services within a framework of continued care for all pregnant women [4, 8].

During this critical prenatal period, women and the fetus face multiple risks that are life threatening to them and could directly impact their survival [9–12]. Effective detection and management of these complications require facility-based, skilled care within antenatal care services [13, 14]. First contact during the first three months of pregnancy (timely), a minimum of four, most recently at least eight contacts for antenatal care (sufficient) and adequate services (with appropriate content) prenatal care service is significantly important in improving maternal and neonatal health outcomes [4, 15]. This indicates that prenatal care could positively influence birth outcomes by producing changes in mother’s behaviors, improving mother’s nutritional intake, reducing morbidity risks and terminating pregnancy that could lead to poor birth outcomes through the application of its four goals: early detection of pregnant women at risk, action to prevent any future difficulties, diagnosis and treatment of preexisting medical conditions, and prompt referral in case of complications developed [16, 17].

Evidence shows that the impact of prenatal care on mothers and newborns depends not only on the mere occurrence of a visit but also on the quantity and quality of the procedures performed at each visit [16]. The WHO and other relevant bodies recommended certain prenatal care packages that need to be provided to all pregnant women at different levels of prenatal care contact (visits). According to the focused antenatal care model that recommends a minimum of four prenatal care visits and content of the packages that comprise physical examinations (blood pressure measurement, fetal heartbeat assessment), laboratory investigations (urine and blood samples), preventive procedures (tetanus injection and iron supplementation) and counseling on signs of pregnancy complications and measures to be taken by the mother [18].

Even though prenatal care service coverage is increasingly available to women in low- and middle-income countries (LMICs), the content, timing, and frequency of antenatal care services are inadequate [1, 2, 19]. According to a study in Addis Ababa, Ethiopia, only 11 % of pregnant women received adequate prenatal care, and 35.4 % started prenatal care in the first trimester in Gondar Town, Ethiopia. This shows significant variation across regions [20]. The disparities could be due to women’s educational status, marital status and planned pregnancy [21, 22]. Furthermore, early visits for prenatal care were also influenced by maternal age at marriage, perceived the right time by the women to start prenatal care, wealth status and decision-making power to use prenatal care [21, 22]. The above evidence did not consider all the three main components to compute prenatal care adequacy, for better impact of prenatal care services on safe motherhood.

 This study was conducted at the community level using a longitudinal approach, and the analysis also included the three recommended contents of prenatal care. This study provides a more representative and comprehensive illustration of prenatal care adequacy in line with the WHO recommendation and factors associated with it in Ethiopia than previous studies that were unidirectional [23–25]. Moreover, Southern nations, nationalities, and people’s region is an extremely ethnically diverse region of Ethiopia, inhabited by more than 80 ethnic groups, of which over 45 (or 56 %) are indigenous to the region [26]. And also universal health coverage in the region is only 27.5 % compared to 52.2 % in Addis Ababa, the capital of the country [27]. Thus, this study aimed to assess the adequacy of antenatal care and associated factors in the Southern Ethiopia.

## Materials and methods

### Data source

The study was conducted in the Southern Nations Nationalities and Peoples Region of Ethiopia, between August 2016 and January 2017. It expanded on the previously implemented Performance Monitoring and Accountability 2020 (PMA2020) survey, a longitudinal data (following pregnant women through one year postpartum).

It is available from https://www.pmadata.org/countries/ethiopia.

### Study population and sampling

A total of 328 households had at least one woman who met the study eligibility criteria. A total of 329 women met the study eligibility criteria and were enrolled in the study (one household had two pregnant women enrolled in the study). All women who were pregnant for six months or above were included.

Forty-four enumeration areas (EAs) used in PMA2020/Ethiopia were included in the sample. A multistage stratified cluster design in which all EAs were randomly selected using probability proportional to size and all households were screened to identify any women six or more months pregnant. All consenting pregnant women were interviewed at screening, seven days, six weeks, and six months postpartum by trained enumerators using smartphones programmed with Open Data Kit (ODK) [28].

## Measurement of variables

### Response variable: prenatal care adequacy

The overall prenatal care adequacy indicator was constructed using the three prenatal care utilization indicators, that is early prenatal care (the first prenatal care visit made during first trimester), enough visits (at least four prenatal care visits) and sufficient services (all core services performed at least once during the pregnancy care) [29]. To assess service content, participants were asked about the basic prenatal care components received as recommended by the WHO for all women regardless of the gestational age at the first visit to clinics [30]. Information on mother’s blood pressure, urine, stool and blood sample taken (blood type, hemoglobin (anemia) and syphilis test), tetanus injection, iron supplementation, and information or counseling given about signs of prenatal care complications (abdominal pain, severe headache, vaginal bleeding), and post-partum family planning was obtained from respondents. Service content was categorized as sufficient if all the above services were provided to the mother according to the national recommendation, at least once during the last prenatal care, otherwise insufficient. Finally, prenatal care was defined as adequate if the woman had attended prenatal care early; enough visits and sufficient services; otherwise, inadequate.

#### Explanatory variables

The independent variables for this study were selected among the variables used by PMA2020 for data collection matching them with predisposing factors and enabling factors described in “Andersen’s Health Care Utilization Model” [31], including age, parity, educational status, occupation, ethnicity, religion, marital status, residence, relationship to head of household, number of children, wealth, membership status of women in one to five networks and maternal characteristics such as place prenatal care and service providers.

### Statistical analysis

Bi-variable and multivariable regression analyses for ordinal responses were employed to assess the factors associated with prenatal care adequacy. The generalized ordered logit model estimates the odds of being beyond or below the level of the dependent variable, and the variables exert the same effect on each cumulative logit regardless of the cut off value for prenatal care adequacy. The classic ordinal proportional odds model cannot be applied if one predictor variable violates the proportional odds assumption. The Brant test is typically used to determine whether the assumption holds, but because the survey data are weighted, the Wald test is more appropriate. Therefore, each variable was first tested individually to determine whether the requirements of the proportional odds assumption were satisfied. Weighted results are reported to account for the PMA2020 sampling design, and the variances of the covariates were adjusted accordingly. Data analysis was performed using STATA statistical software SE version 16.0 (Stata Corp. College Station, TX).

### Ethical considerations

PMA2020 survey activities received prior ethical approval certified through Ethiopia’s and John Hopkins University’s Institutional Review Board system, and participants were consented to participate in the interviews. Approval for access to the anonymized data at the time of study conception was granted by Maastricht University under registration number FHML/GH_2019.093. As a secondary analysis using exclusively anonymized data, this study was determined not to qualify as a human subject’s research and waived the requirement for informed consent.

## Results

### Sociodemographic characteristics of the respondents

A total of 324 women were interviewed for the study, yielding a response rate of 98.5 %. The mean (± SD) age of the women was 26.23 (+ 5.76) years. As Table 1 shows, 57.36 % of the women were urban by residence. The educational status of 40.55 % respondents was primary school, followed by 29.27 % who never attended formal education and 17.38 % who attended secondary school. Nearly half (45.05 %) of the participants had 2 to 4 children, with the average (± SD) number of children being 3.07 (+ 2.15) (Table 1).

**Table 1:** Sociodemographic variables of women of reproductive age in Southern Ethiopia (n = 329), weighted

Socio-demographic characteristics	Category	Weighted Frequency	Weighted Percent
Age group (in years)	15–24 years	108	32.8
	25–34 years	170	51.8
	35–49 years	51	15.4
Residence	Urban	37	11.3
	Rural	292	88.7
Household wealth	Poor	123	37.4
	Middle	110	33.5
	Rich	96	29.1
Educational status^+^	Never attended	146	44.9
	Primary	144	44.3
	Secondary/technical/higher	35	10.8
Marital status	Married	319	97.1
	Unmarried	10	2.9
Parity^+^	1	67	20.7
	2–3	82	25.4
	4 or more	175	54.0

+ Data collected at 7-day postpartum, n = 324

### Maternal characteristics

Of the total respondents, 82.9 % attended at least one prenatal care in the study area. As Table 2 shows, 30.2 % of the respondents received prenatal care from health extension workers only by the source of prenatal care services. The place of prenatal care follow-up was 58.4 % at public health facilities, followed by 25.6 % health posts and 8.6 % at public hospitals (Table 2).

**Table 2:** Maternal characteristics of the respondents in southern Ethiopia

Maternal characteristics	Category	Weighted Frequency	Weighted Percent
Received prenatal care	Yes	269	82.9
	No	55	17.1
Source of prenatal care	HEW only	81	30.2
	OHP only	78	28.8
	Both HEW and OHP	110	40.9
Place of antenatal care	Home	16	5.9
	Public hospital	23	8.6
	Public health center	157	58.4
	Health post	69	25.6
	Private hospital	4	1.5
Participation in 1–5 group	Yes	50	18.5
	No, member but did not participate	52	19.5
	No, not a member	167	62.0

*NGO * nongovernmental organization, *HEW*  health extension workers, *OHP *other health professionals

### Adequacy of prenatal care

More than half (52.6 %) of the women who received prenatal care services received sufficient visits, while only 11.2 % had an early attendance. As Table 3 shows, 57.9 % of those who attended prenatal care services did not receive sufficient services based on the content of services.

**Table 3:** Proportions of mothers who received core components of adequate prenatal care services in southern Ethiopia, weighted n = 269

Variables	Category	Weighted Frequency	Weighted Percent
**Frequency of prenatal care**	Less than four visits	127	47.4
	Four and above visits (enough visits)	142	52.6
**First visit of prenatal care**	Less than 12 weeks (early attendance)	30	11.2
	12 weeks and above	239	88.8
**Received sufficient services**	No	156	57.9
	yes	113	42.1

### Proportions of care received during pregnancy by the respondents

**Fig. 1:**
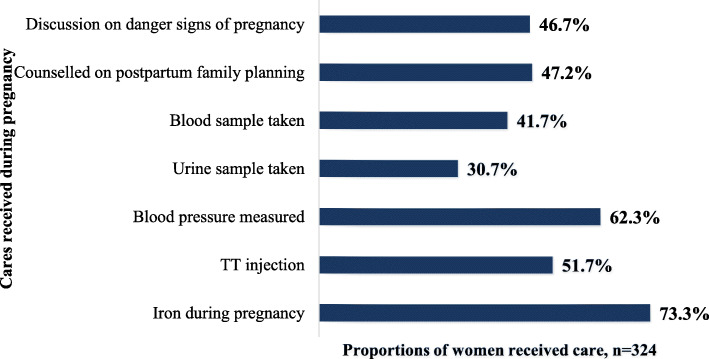
Proportions of women who received sufficient antenatal care services among respondents in South Ethiopia

The most common services received by the respondents during pregnancy were iron (73.3 %), followed by blood pressure (62.3 %) and TT injection (51.7 %), while the least common service received prenatal services (30.7 %) (Fig. 1).

**Fig. 2:**
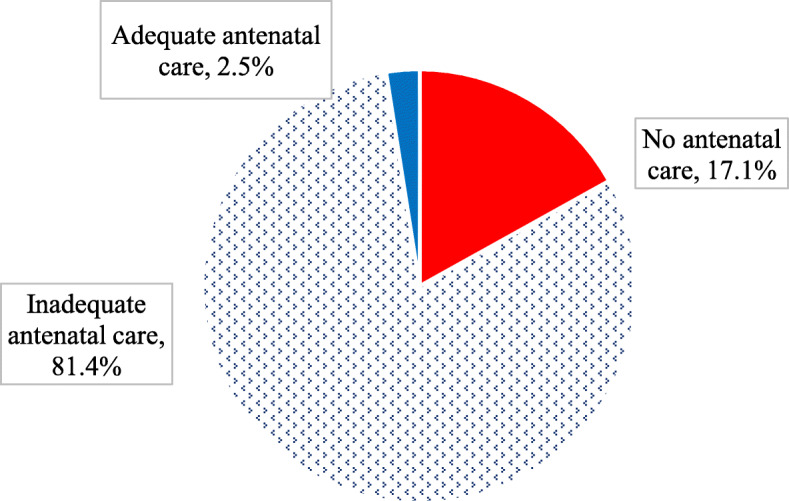
Overall adequacy of prenatal care among respondents in South Ethiopia

Overall, only 2.5 % of the women received adequate prenatal care services, whereas 17.1 % had no antenatal care (Fig. 2).

### Factors influencing adequacy of prenatal care in Southern Ethiopia

Multivariable ordinal logistic regression revealed that residence, women one to five networks, and educational status were found to be statistically significant factors associated with adequacy of prenatal care in Southern Ethiopia.

Urban women had twice (aOR 2.35, CI. 1.05–5.31) more likely to receive adequate antenatal care than rural care by residence. Women who participated in one to five female networks were approximately three times (aOR 2.78, CI. 1.01, 7.71) were more likely to receive adequate prenatal care than their counterparts.

Educational status was also found to be a significant factor even after controlling for other confounders. Respondents who attended primary level of education were more than twice (aOR 2.42 95 % C.I. 1.04, 5.65), and those who had completed at least nine years of schooling were four times (aOR 4.18 95 % CI. 1.32, 13.29) more likely to utilize adequate prenatal care than those who did not attend formal education (Table 4).

**Table 4:** Ordered logistic regression of prenatal care adequacy in Southern Ethiopia

**Variables**	**Odds ratio**	**Coef.**	**P-value**	**[95% C.I]**	
**Residence**					
Urban	2.35	.858	0.038	1.05	5.31
Rural	1.0			1.0	
**Member of 1x5 women network**					
Yes	2.78	1.024	0.049	1.01	7.71
No					
**Educational status**					
Primary	2.42	.886	0.040	1.04	5.65
Secondary and Above	4.18	1.431	0.015	1.32	13.29
**Number of Children**	.95	-.052	.560	.79	1.13

### Discussion

This study is comprehensive in addressing all the dimensions of prenatal care contents to show the particular care being received by pregnant women and to indicate the magnitude of each component of care in the southern region of Ethiopia. The study results help to recommend specific gaps and general factors associated with the adequacy of services. Research has tended to focus on single measurements, more specifically the prevalence of service utilization and timing of prenatal care [24, 25], rather than adequacy of prenatal care. Thus, this study combined the indicators of “early visits, enough visits, and sufficient services of prenatal care” indexes to construct adequacy of prenatal care as per the WHO recommendation [32].

As we can see from this study’s results, the majority (84.10 %) of the study participants initiated prenatal care within the first trimester (before 12 weeks of gestational age), less than half (44.21 %) of them had four or more visits, and few (42.03 %) of them received sufficient services. This indicates that the initiation of prenatal care has better coverage compared to the remaining two prenatal care contents in Southern nations, nationalities and peoples’ region of Ethiopia, which suggests additional activities and interventions on the remaining two dimensions. Moreover, the finding indicates that the problem that affects the adequacy of prenatal care service was higher from the providers’ side compared to the receivers’ side because the utilization rate decreased after the first visit, where the sufficiency of the provided service is low.

The single most conspicuous observation to emerge from the data was the 2.45 % prevalence of prenatal care adequacy. We found much lower values (11 %) than a study in Addis Ababa Ethiopia among slum populations [33]. The variation can be due to the difference in study setting; Addis Ababa is the largest city with a better literacy rate, lifestyle, income, access to health information and health facilities.

The association between prenatal care adequacy and women’s residence is worth mentioning because women living in urban areas were more likely to receive adequate prenatal care than their counterparts. This is in good agreement with the further analysis of Ethiopian demographic and health survey data in 2016 and in the Benishangul Gumuz Region of Western Ethiopia, which revealed that place of residence was associated with utilization of at least four prenatal care visits [24, 34]. This implies that health service inequity is avoidable and unfair systematic differences in the health of populations rooted in social determinants of health [35]. Therefore, health equity and social determinants become the critical components of the post millennium goal and sustainable development global agendas as a part of progressive achievement for universal health coverage [8]. The social determinants of health, such as the conditions in which people are born, grow, work, live, and age, and the wider set of forces and systems, such as economic policies and systems, development agendas, social norms, social policies and political systems, which shape the conditions of peoples’ daily life [36], bring health inequity.

 This study found that better women’s educational status was significantly associated with the adequacy of prenatal care services. This substantiates previous findings in the literature, in Addis Ababa Ethiopia, showing that less educated women had lower overall adequate use of prenatal care [21]. Additionally, a support study conducted in the Hadiya Zone, Southern Ethiopia and a study in Western Ethiopia identified that maternal education was a major predictor of antenatal care service utilization [37, 38]. Likewise, mothers’ education level matters the frequency of prenatal care utilization by pregnant women in Ethiopia to the large and in Tabor Town, northwest Ethiopia [23, 34].

 Our study provides considerable insight into the association of prenatal care adequacy and women’s one-to-five networks for discussion of family health issues. Members of the one to five networks were found to receive higher adequacy of prenatal care service. This is consistent with a study undertaken in four regions of Ethiopia, including the southern region, which revealed that women’s participation in one to five women’s network was associated with four or more antenatal care visits [39]. Furthermore, a systematic review of evidence that focused on identifying the contribution of women’s development army to maternal and child health in Ethiopia also indicated that participation and membership of the group led to the use of the prenatal care and delivery service at a higher rate [40].

### Strengths and limitations of the study

The study involved data from different sources using community-based interview of the women, and their records in health facilities improve strength of the evidence. However, the study might be affected by recall bias for the services they received during early prenatal period as a limitation.

### Directions for future research, practice and policy

Policy ensuring adequate prenatal care services for each pregnant mother should be in practice. Public health interventions should give priority for those far to reach areas, and illiterate women, who disproportionately affected by morbidity and mortality. Further study were recommended to validate the tools and measure the adequacy of the services in different contexts of Ethiopia using a mixed method study for an in-depth understanding of the problem.

## Conclusions

Adequacy of prenatal care services in Southern Ethiopia is very low. Fewer than three mothers out of 100 received adequate prenatal care services in Southern Ethiopia. The Ethiopian health care system should strengthen the women’s one to five networks for discussion on family health issues. Moreover, much have to be done on improving the adequacy of prenatal care, especially in rural areas, and women with no formal education.

## Data Availability

The original data from the survey is available from the corresponding author in a Stata™ software.

## Abbreviations

aOR: Adjusted odds ratio
EAs: Enumeration areas
FANC: Focused antenatal care
LMICs: Low- and middle-income countries
ODK: Open Data Kit
PMA: Performance Monitoring and Accountability
WHO: World Health Organization
